# Unusually short VA time during Para‐His pacing maneuver: Is it a simultaneous atrial capture?

**DOI:** 10.1002/joa3.12603

**Published:** 2021-07-21

**Authors:** Debabrata Bera, Sanjeev S. Mukherjee, Rakesh Sarkar, Ruchika Rao, Suchit Majumder

**Affiliations:** ^1^ Department Cardiology RTIICS Kolkata India; ^2^ Department of Cardiology Medical Hospital Kolkata India; ^3^ Department of Cardiology Medanta The Medicity Gurugram India; ^4^ Department of Anaesthesia Medanta The Medicity Gurugram India; ^5^ Department of Cardiology Apollo Gleneagles Hospital Kolkata India

**Keywords:** Para‐His pacing, simultaneous atrial capture, SVT maneuvers, short VA time

## Abstract

Although a very VA interval (<60 ms in proximal CS) is suggestive of simultaneous atrial capture, rarely it can have exception. A very short VA shall not be discarded without analysing the electro grams.

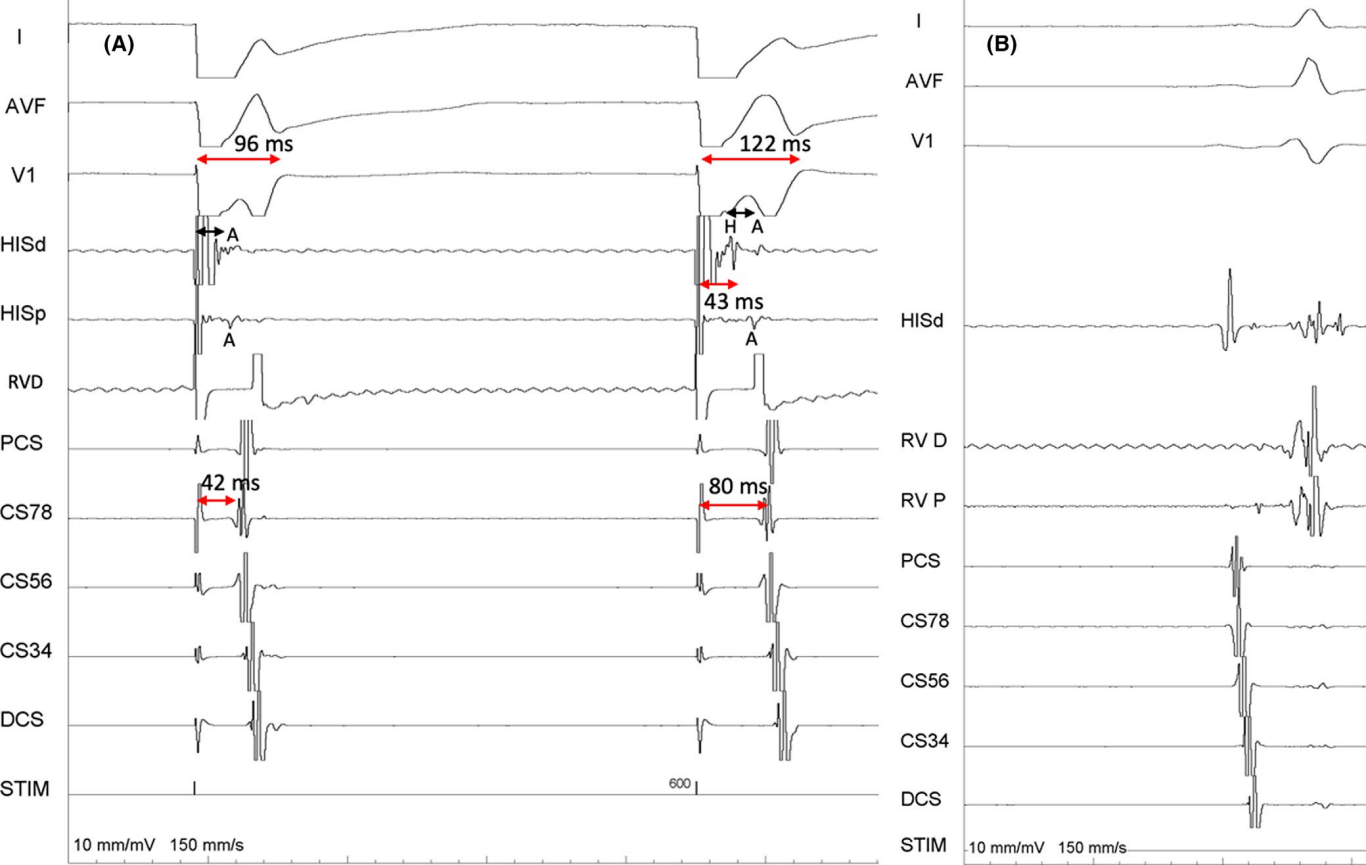

## 1 CASE

**FIGURE 1:**
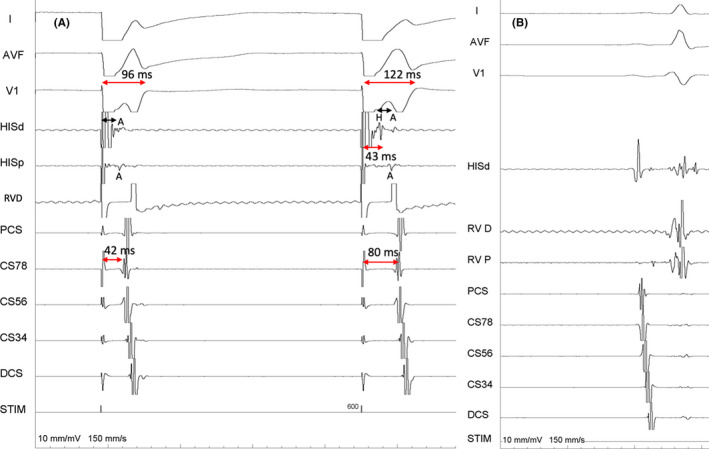
(A) The two complexes during para‐his pacing from the Map‐D pole. The first beat was narrow (96 ms) with His and RV myocardial capture. The second beat on the right side demonstrated a wider QRS duration (122 ms). Moreover, His signal was released during this beat, a definite evidence of only myocardial capture. The Stim‐H (=VH) got prolonged by the same magnitude as SAT (38 ms). The HA remained identical in both beats (28 ms, black arrow) supporting true VA conduction in both beats without SAC. (B) Sinus beat showing short AH (=35ms). The HV interval was 36 ms

A 45‐year‐old lady presented with recurrent narrow QRS short‐RP tachycardia. Baseline ECG was normal with no evidence of pre‐excitation. Echocardiography was unremarkable. During her electrophysiology study, Para‐Hisian pacing (PHP) maneuver was performed before induction of tachycardia. Both the RV catheter and His catheter were placed at His bundle (HB) region (Appendix 1). Pacing at variable output at fixed cycle length was performed from RV distal (RVD) bipole. It was started at an output of 10 mA at a pulse width of 2 ms and the output was gradually reduced till there was widening of paced QRS complex suggestive of loss of HB capture. The measurement of Stim‐to‐A time interval (SAT) recorded in the proximal coronary sinus (PCS was positioned at CS ostium) bipole was only 42 ms (Figure 1A).

What is the mechanism behind such a short SAT? Is it due to inadvertent simultaneous atrial capture at the high output (hence making the maneuver uninterpretable) or could this be a genuine VA conduction suggestive of nodal VA conduction?

## 2 COMMENTARY

Para‐Hisian pacing (PHP) is an extremely useful maneuver in differentiating the route of VA conduction via septal accessory pathway versus AV node. Hence, it has a crucial role in supporting the diagnosis of AVRT versus atypical AVNRT, respectively. Moreover, it can help in mapping of septal concealed accessory pathways (AP) during RV pacing. However, there are a few pitfalls of this maneuver unless performed appropriately. It can only show the presence of VA route (ie, AP/node) in sinus rhythm, but cannot prove participation of the pathway/node during tachycardia. Moreover, PHP, is often misleading for slowly conducting accessory pathways or free wall APs, where conduction up the pathway is often masked by faster conduction up the AV node, if concomitantly present.

Another major pitfall during PHP is inadvertent simultaneous atrial capture (SAC) at higher output. Generally, it is accepted that if VA timing is very short, it is not physiological to have a genuine VA conduction. The only paper looking for a cut‐off value, by Obeyesekere et al^1^ has concluded that Stim‐A time (SAT) in PCS <60 ms as definite evidence of inadvertent atrial capture. On the contrary, SAT is more than 90 ms is unequivocally an evidence of true VA conduction. A SAT of 60‐90 ms can be either way, which needs to be carefully analyzed. “A‐EGM’ (electrogram) in His catheter (His‐A) can be a better indicator, but often have practical difficulty due to saturation artifact unless 2 catheters are placed near HB region.

In this case, we encountered an exception to this rule. The SAT was very short around 42 ms “mimicking” a SAC. The retrograde *atrial activation sequence* was confirmed to be the *same* during only V and H + V capture confirming only one route for the VA conduction. Then, PHP was repeated multiple times with slight adjustment of the pacing catheter (RVD) in the His region. Every single time the SAT was initially very short and it prolonged reproducibly and exclusively with concomitant widening of QRS complex. It was proved to be a genuine VA conduction as discussed below:

First, only during a true nodal response of VA conduction, the SAT would increase with concomitant widening of QRS. If the SAT prolongation was due to loss of SAC, then there would be no change of QRS morphology. In our case, the widening of SAT reproducibly happened during simultaneous QRS widening suggesting true VA via AV node.

Second, the EGMs in His‐D bipole provide key inputs to the case. We notice three potentials in His‐D. During V capture in second beat the sequence is certainly V‐H‐A. The third signal is therefore A‐EGM. The second signal after V‐EGM is His signal which was released in the wider second beat supporting only myocardial capture. This happened reproducibly. Still, there might be an extremely rare theoretical possibility where the transition from H + V capture to only V capture and SAC to true VA changeover happened in exactly the same beat. The HA time remained fixed around 28 ms in both beats again supports true VA over SAC. Had it been a SAC the A in first beat would have occurred with pacing spike so the HA would have been zero unlike here.

Finally, a maneuver can be of additional help in cases of suspected SAC. If ventricular extra stimulus (VES [S1‐S2]) protocol is performed from the pacing catheter in cases with SAC the SAT will remain same, whereas in genuine VA, the SAT will decrement in a nodal conduction. However, if VA conduction is via accessory pathway, the SAT may remain the same even during VES. It was although not performed in our case.

To summarize, from the initial two points we proved the first beat as true VA conduction despite having very short VA time. The Delta‐SAT in PCS was 38 ms, which was nearly the same to the increment of VH (delta‐VH is 38 ms [43‐5 ms]; assuming a Stim‐to‐H during the first beat of approximately 5 ms).

We pondered about the possible causes of such a short SAT. One possibility could be a robust AV node and the supra‐His circuit having brisk conduction. We believe in our case the retrograde AV nodal conduction via fast pathway was brisk like her antegrade conduction (short AH (=35 ms) in sinus rhythm [Figure 1B]). This could be related to increased sympathetic tone in the patient during the procedure. Here, in addition, the SAT in PCS was short too, with near simultaneous His‐A and PCS‐A timing. We hypothesize this could be due to the breakthrough of fast pathway retrograde conduction midway between His‐D and PCS. To complete the case, our patient had a typical slow‐fast AVNRT which could be readily cured with slow pathway modification in the lower triangle of Koch. This case is an exception to the only paper looking at the cut‐offs of SAT for particularly determining inadvertent atrial capture.

## CONFLICT OF INTEREST

The authors declare no conflict of interests for this article.

## APPENDIX 1

RAO (30°) and LAO (40°) cine image showing both His and RV catheter near the HB region. The PHP was performed from RV distal bipole.
